# Gait pattern stratification in persons with incomplete spinal cord injury: a data-driven approach

**DOI:** 10.1186/s12984-026-01896-w

**Published:** 2026-02-26

**Authors:** Minh Tat Nhat Truong, Emelie Butler Forslund, Åke Seiger, Elena M. Gutierrez-Farewik

**Affiliations:** 1 KTH MoveAbility, Department of Engineering Mechanics, KTH Royal Institute of Technology, 100 44 Stockholm, Sweden; 2 Aleris Rehab Station R&D Unit, 169 89 Solna, Sweden; 3 Department of Neurobiology, Care Sciences and Society, Division of Physiotherapy and Division of Clinical Geriatrics, Karolinska Institutet, 141 83 Stockholm, Sweden; 4 Department of Neurobiology, Care Sciences and Society, Division of Clinical Geriatrics, Karolinska Institutet, 141 83 Stockholm, Sweden; 5 Department of Women’s and Children’s Health, Karolinska Institutet, 171 77 Stockholm, Sweden

**Keywords:** Multivariate time series, Hierarchical clustering analysis, Pathological gait, Binary classification, Shapley addictive explanations

## Abstract

**Background:**

Incomplete spinal cord injury (iSCI) often causes heterogeneous locomotion dysfunctions, depending on remaining sensorimotor function. Clinical tests and traditional gait analysis have limited ability to quantify the diversity of gait impairments. Unsupervised learning techniques can objectively identify common gait patterns among the overall heterogeneity. Explainable artificial intelligence approaches, when combined with machine learning models, can reveal important features often missed by traditional gait analyses. This study presents a framework to characterize gait heterogeneity among persons with iSCI based on several data-driven methods. We aimed to stratify overall gait heterogeneity by deriving clusters with similarities without a priori identification of parameters, and to assess possible clinical correlations in the derived clusters.

**Methods:**

A cohort of 28 adults with iSCI and control group of 21 non-disabled adults were recruited. The iSCI group underwent a standard physical assessment of overall mobility, lower extremity strength, and spasticity. Both groups underwent instrumented 3D gait analysis, walking at self-selected pace. Distinct iSCI gait pattern subgroups were identified with dependent dynamic time warping and hierarchical agglomerative clustering. Distribution of clinical descriptives and outcome measures among and between groups were evaluated. Gait predictors that distinguish each cluster from control gait were identified with a random forest classifier and explainable AI.

**Results:**

Six distinct gait clusters were identified among the 280 iSCI gait cycles. Clusters with relatively low walking speed exhibited shorter step lengths and less ankle plantarflexion in pre-swing than controls. Gait patterns and walking performance in clusters with high walking speed were relatively similar to controls. Overall muscle strength, walking independence, walking speed, step length, step width, sex distribution, and types of walking aids significantly differed between all six clusters. Ankle plantarflexion angle in pre-swing correlated strongly with walking speed and step length.

**Conclusion:**

Through a series of advanced data-driven approaches, common gait patterns can be objectively identified and comprehensively characterized within a heterogeneous iSCI population. This work represents an initial step in developing individualized rehabilitation programs for persons with iSCI.

## Background

Spinal cord injury (SCI) leads to partial or complete loss of sensory and/or motor neural functions in neural levels at or below the level of injury. Changes in functional ambulation category can reflect neurological recovery and adaptation after SCI. The American Spinal Injury Association (ASIA) Impairment Scale (AIS) [1, 2] as instructed by the International Standards for Neurological Classification of SCI is recommended to classify injury severity. Persons with AIS A, or complete SCI, commonly have limited recovery potential, with approximately 5% reaching community-level ambulation [3]. Incomplete SCI (iSCI) ranges from AIS B to E, with increasing order of regaining ambulation after injury with rehabilitation [4]. While the AIS scale provides useful clinical information and recovery prognosis, it inadequately captures ambulatory gains in people with SCI [5], necessitating more sensitive assessment tools.

Ambulatory capacity in the overall population with iSCI is very heterogeneous, with ambulation level ranging from no independent walking without full assistance to practically normal function [6, 7], thus predicting walking function after rehabilitation and/or therapy is not straightforward. Current standardized clinical assessments have limitations in evaluating gait function: Lower Extremity Motor Score [1, 8] and Manual Muscle Test [9] are semi-quantitative grades and rely on subjective strength evaluation; Walking Index for Spinal Cord Injury (WISCI II) [10] lacks sensitivity for mild iSCI; and while the Ten-meter Walk Test (10mWT) [11] and Six-Minute Walk Test (6MWT) [12] assess basic locomotion performance and endurance, they lack comprehensive kinematic and kinetic information and have limited use in personalizing rehabilitation programs.

Gait and motion analysis provides comprehensive and quantitative characterization of neuromuscular deficits and compensatory movement patterns, including clinically meaningful insights of gait impairments in iSCI. Important insights have been discovered: range and rate of knee flexion/extension correlating with injury level [13]; longer cycle duration with increased hip/knee flexion during treadmill walking [14]; varying walking speed, ranging from 0.3 m/s to 1.05 m/s [15]; and greater minimum margin of stability indicating lower stance stability [16]. These findings of isolated temporospatial parameters and/or kinematics features complement clinical assessments, but do not fully provide a holistic picture of an individual’s gait function and of the heterogeneity among the patient population.

Unsupervised learning algorithms such as hierarchical clustering or k-means clustering can reveal subgroups within a dataset without requiring prior domain knowledge or bias toward particular parameters [17–19]. Two notable studies have applied clustering algorithms to SCI populations; Basiratzadeh et al. used spectral clustering on 334 SCI individuals’ baseline variables (injury location, severity, Functional Independence Measure motor score, length of stay, and demographics) [20], identifying 5 distinct clusters, though gait differences between clusters were not analyzed. Werner et al. applied k-means clustering to inertial sensor data from 66 iSCI individuals [21], identifying 4 clusters based on gait features, but without direct analysis of kinematics cycles. These data-driven techniques demonstrate the potential of unsupervised clustering algorithms in SCI populations.

Dynamic time warping (DTW) enables fair comparison of multidimensional time series data by accounting for temporal misalignments between potentially temporally mismatched cycles [22–26]. Combined with clustering methods, DTW has effectively identified subgroups in various domains: human DNA [27], electrical household load [28], and gait characteristics [29, 30]. Recent applications include characterizing gait heterogeneity in non-disabled individuals [31] and in childen with hereditary spastic paraplegia [32]. Thus, DTW has potential for better clustering results, especially on 3D gait patterns.

Stratifying gait characteristics requires more than simply clustering gait patterns; extracting clinically meaningful features and exploring their relationships to cluster properties can provide critical insights. Machine learning models can reveal complex relationships between gait features and cluster patterns beyond classical linear regression [33]. When trained on a predefined dataset, machine learning algorithms can capture the underlying structure of different profiles without requiring the relationships between input features and cluster labels to be explicitly defined. Tree-based models like random forests [34] can capture nonlinear relationships through ensemble predictions of decision trees, generating impurity-based feature importance values of its input feature variables. One drawback of this impurity-based feature importance algorithm is that it can only provide average effects across the dataset. To this end, model explanation techniques built specially for tree-based models such as TreeSHAP [35] can provide conditional feature effects for individual predictions and rank the contribution of each feature in tree-based model decisions through Shapley values [36]. As they provide insights into features’ contributions to the predictions of the tree-based model, the explanations from TreeSHAP are particularly useful in various domains such as breast cancer diagnosis [37], heart-related research [38, 39], and satellite-based prediction of precipitation [40].

The aims of this study are two-fold: (i) to stratify gait patterns among a study population with iSCI into similar subgroups; (ii) to gain clinical insights into derived subgroups. The study design is a case–control cross-sectional study.

## Methods

### Subject recruitment

In this study, we recruited adults with incomplete paraplegia due to SCI (iSCI group) and a cohort of non-disabled adults (control group) for reference. The inclusion criteria for the iSCI group were: (i) age 18–80 years; (ii) habitually walking in daily life; (iii) able to walk at least 30 m with or without technical aids or orthoses; (iv) full neurological function in the arms; (v) at least 1 year post injury or considered neurologically stable; (vi) cognitive and cooperative ability to complete the entire protocol. The exclusion criteria were any other conditions in addition to SCI that may affect gait. The inclusion criteria for the control group were: (i) age 18–80 years; (ii) no known pathology or recent injuries that affect walking ability.

We screened a total of 37 participants with SCI. We adjusted our protocol after screening the first three pilots (S1, S2, S3), so we excluded their data in this study. Three other participants (S8, S10, S14) were unable to fully complete the data collection due to health issues. Moreover, we also screened three more participants (S12, S13, S34) but subsequently excluded their data because they presented with either complete motor (S13 & S34) or complete sensory (S12) injury. After exclusions, data from 28 participants with iSCI were included in this study. We also recruited a convenience sample of 21 non-disabled participants for our control group.

The study was approved by The Swedish Ethical Review Authority (Diary No.: 2020-02311, 2020-07067, and 2022-00629-02). All participants were fully informed about the data collection protocol and gave their written consent before starting the data collection. The participants could withdraw their participation at any time during the study without stating any reasons. All the measurements were conducted following the standards of Declaration of Helsinki and Good Clinical Practice.

### Data collection protocol

The protocol in this study is part of a comprehensive study on individuals with SCI [41]. An experienced physiotherapist performed all clinical evaluations of the participants with iSCI at the Spinalis Unit, Aleris Rehab Station, Stockholm, Sweden. Each participant with iSCI was first classified based on AIS score and overall mobility was graded as WISCI II scores. Sensory Level of Injury (SLI) is determined by the most caudal (lowest) dermatome showing normal response to both pin prick and light touch testing. Motor Level of Injury (MLI) is determined by the lowest key muscle scoring at least 3/5 in strength testing, given all key muscles above that level being evaluated as intact [1]. Lower limb motor function was evaluated with MMT scores. Spasticity in major lower limb muscle groups was evaluated with a modified Ashworth scale [42] and noted as absent (score 0) or present (score 1-4).

**Fig. 1:**
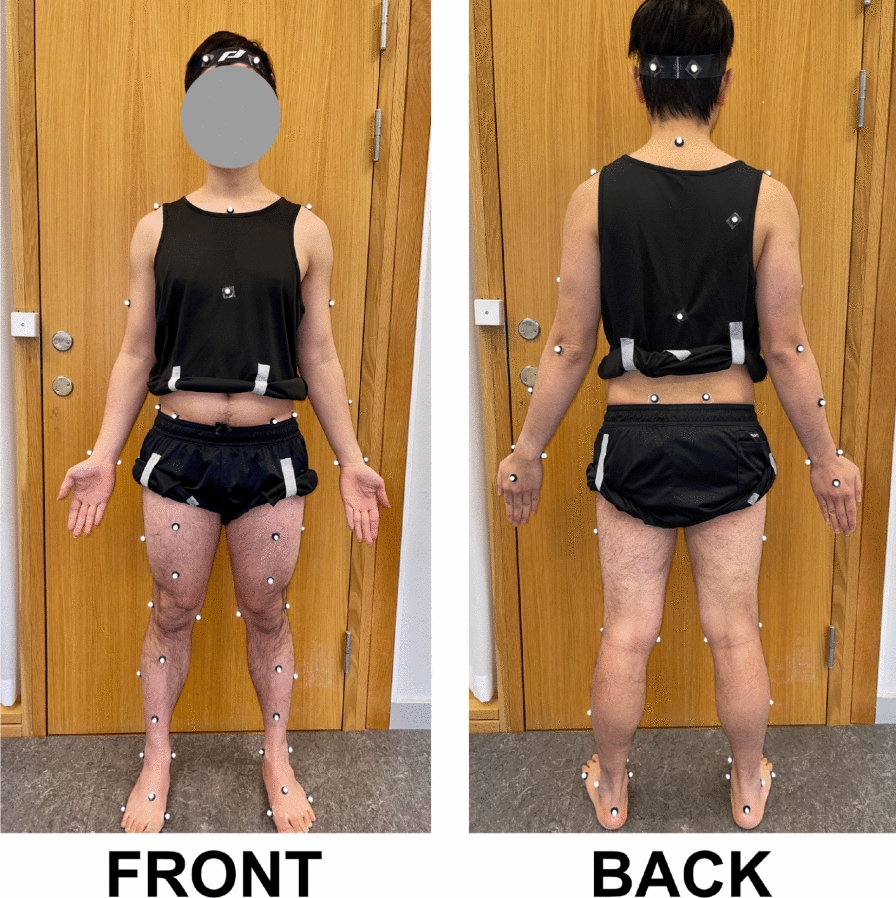
Marker placement according to the 71 markers in the CGM2.4 protocol plus two additional tracking markers on the pelvis. The subject consented for the image to be freely available on the internet and seen by the general public

All participants underwent instrumented 3D motion analysis at the Promobilia MoveAbility Lab, KTH Royal Institute of Technology, Stockholm, Sweden. Anthropometrical parameters were measured, and reflective markers were attached on body landmarks according to the CGM2.4 marker set [43, 44] (Figure 1). Marker trajectories were captured (10 Vicon V16 cameras, Oxford, UK) at 100 Hz. After a static trial, data was collected during gait along an instrumented walkway at a self-selected comfortable speed. Participants in the iSCI group were encouraged to walk barefoot or with as few assistive devices as possible. All participants in the control group walked barefoot. In all conditions and participants, data from several gait cycles from each side were collected from which 5 cycles per side were used for further analysis. The two sessions were conducted on two different days.

### Data post-processing

In the iSCI group, the stronger leg (SL) and weaker leg (WL) were determined based on sum MMT scores from each leg. In cases of equal sum MMT scores, WL was determined based on summed joint MMT scores in the order hip, knee, ankle, then hallux. If leg and joint MMT sum scores were equal, the right side was chosen as the WL. When the therapist scored with a plus or minus, these were quantified as 0.5; e.g., a grade 3+ was quantified as 3.5 and a 3-, as 2.5.

Marker trajectories were gap-filled and filtered (Vicon Nexus 2.12, Oxford, UK), and kinematics were computed according to the CGM2.4 model [43]. For each gait cycle, kinematics time series from nine degrees of freedom (DoFs) was used for further analysis: pelvis tilt, pelvis obliquity, pelvis internal/external rotation, hip flexion/extension, hip ab/adduction, hip internal/external rotation, knee flexion/extension, ankle dorsi/plantarflexion, and global foot progression angle, corresponding to frequently analyzed gait kinematics [45, 46]. Temporospatial parameters, specifically walking speed, step length, and step width, for each gait cycle were analyzed, wherein step width was defined by the mediolateral distance between the heel markers at initial contact.

### Cluster identification with dynamic time warping and hierarchical agglomerative clustering

**Fig. 2:**
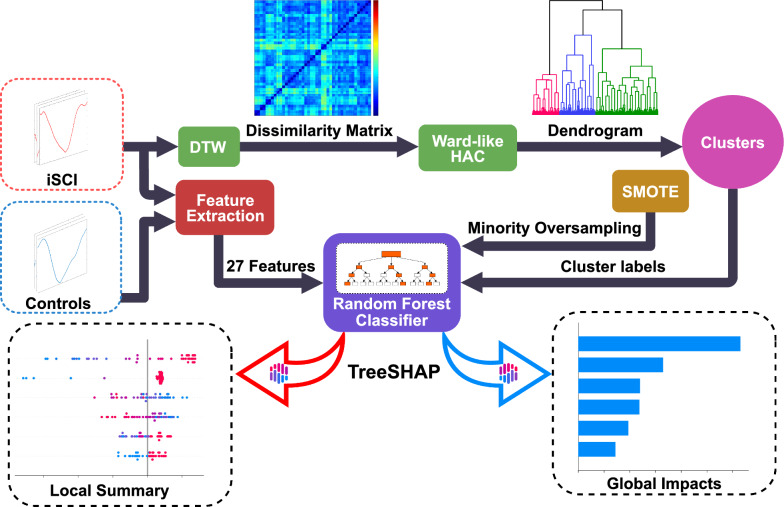
Illustration of the process to stratify the heterogeneous gait patterns in our study population with iSCI. Each 3D gait pattern consists of 9 kinematics time series. Gait patterns were grouped into clusters with hierarchical agglomerative clustering (HAC) using Ward-like linkage and dependent dynamic time warping ($\mathrm {DTW_D}$) distances between patterns. We then identified features that best separate each cluster from the control group gait patterns. For *n* identified clusters were identified, there would be *n* models. A dataset was constructed for each model, with inputs consisting of 27 selected kinematics features per gait pattern. To balance the groups before classification, the dataset was augmented using the synthetic minority oversampling method (SMOTE). On this augmented dataset, a Random Forest Classifier was trained and cross-validated to determine optimal hyperparameters yielding highest accuracy. The optimal model was then explained using TreeSHAP to show the features that were most important in distinguishing the cluster of interest from the control group. Global impacts indicate the overall importance of features based on average absolute SHAP values across all gait patterns. Local summary reveals the distribution of individual SHAP values, showing the contribution of each feature to the model’s prediction on specific gait patterns

DTW was used to measure gait dissimilarity (Figure 2). DTW was chosen instead of Euclidean distance for the following reaons: Euclidean distance is useful to measure dissiimilarity between time series that have identical lengths that contain features with identical timing. DTW, on the other hand, can measure dissimilarity between two time series that have similar characteristics but contain temporal shifts and/or different lengths. Given the uniqueness of any gait pattern, DTW can perform better in clustering tasks by picking up shared hidden trends among investigated gait patterns while being insensitive to differences in gait events. DTW can also compare raw gait patterns in time domain without the need of normalizing them to gait percentages, meaning that temporal properties of gait kinematics can be preserved and contribute to the clustering process. Literature also showed that clustering algorithm worked better with raw kinematics data [31]. Regarding multidimensional time series data, there are generally two methods of computing DTW depending on the existence of correlations between the dimensions: independent DTW ($\mathrm {DTW_I}$) or dependent DTW ($\mathrm {DTW_D}$) [25]. $\mathrm {DTW_I}$ finds optimal paths corresponding to each dimension while there is only one global optimal path for all dimensions in $\mathrm {DTW_D}$. Since the dimensions were tightly coupled in this study, we chose $\mathrm {DTW_D}$ to measuring the dissimilarities between gait patterns.

For more accurate measures of gait dissimilarity, Z-normalization, a process in which the values of the dataset are rescaled to a standardised scale with a mean of 0 and a standard deviation of 1, was performed in each dimension of all gait pattern before computing DTW [25] to remove biases toward dimensions in the sagittal plane. Specifically, we computed one global mean $\mu$ and one standard deviation $\sigma$ for each dimension across all iSCI gait patterns and used them in Z-normalizing the iSCI data. The mean and standard deviation of the control dataset were not used in this step. For each individual kinematics time series $g_i$ of a gait pattern G ($g_i \in G$), the normalized series in the $m^{th}$ dimension ($\tilde{g}_i^m$) was computed as follows:

1 $$\begin{aligned} \tilde{g}_i^m = \frac{g_i - \mu ^m}{\sigma ^m} , m \in [1,2,...,9] \end{aligned}$$

Where $\mu ^m$ is the global mean value in the $m^{th}$ dimension for all gait patterns, and $\sigma ^m$ is the global standard deviation in the $m^{th}$ dimension for all gait patterns.

Detailed description of the algorithm for $\mathrm {DTW_D}$ computation can be found in the literature [23–26]. Here, the $\mathrm {DTW_D}$ distance between any two 9-D Z-normalized gait patterns $\tilde{G_i}$ and $\tilde{G_j}$, was computed as follows:

2 $$\begin{aligned} DTW_D(\tilde{G}_i,\tilde{G}_j) = DTW(w_1\{\tilde{g}_{i1}, \tilde{g}_{j1}\}, ..., w_9\{\tilde{g}_{i9}, \tilde{g}_{j9}\}) \end{aligned}$$

where $w_1$, $w_2$, ..., $w_9$ are weights assigned to each dimension to determine its importance in finding the optimal path for $\mathrm {DTW_D}$, and $\tilde{g}_{im}$ and $\tilde{g}_{jm}$ are individual kinematics time series of $\tilde{G_i}$ and $\tilde{G_j}$ in the $m^{th}$ dimension, respectively. We considered the gait kinematics time series sequences of the left and right sides independently. To reduce the effects of shared pelvis kinematics on each side, we assigned it with half the weight of the hip, knee, ankle, and foot DoFs when computing the $\mathrm {DTW_D}$ to promote the dissimilarity in sides. One important constraint in computing DTW is the window size which limits how far forward or backward the warping path can move in aligning points between $\tilde{g}_i$ and $\tilde{g}_j$. For this study, we set the window size as 0.5 or 50$\%$ of the total lengths in computing $\mathrm {DTW_D}$ so that, for example, the first point of $\tilde{g}_i$ can only be aligned or matched with points in the first half of $\tilde{g}_j$.

After performing pairwise $\mathrm {DTW_D}$ computation in all iSCI gait patterns, a dissimilarity matrix was constructed. We then implemented hierarchical agglomerative clustering (HAC) on the dissimilarity matrix using Ward-like linkage [47]. Briefly, each observation started with its own cluster. The algorithm then merged the two closest clusters in each iteration such that the increase of the pseudo within-cluster “inertia” was minimum. The algorithm then continued until a single “umbrella” cluster was formed. The sequence of the merging process resulted in a hierarchy of nested clusters in the form of a tree structure or a dendrogram. We then selected a specific threshold to “cut” the dendrogram based on visual inspection of the dendrogram and of the clusters/branches below the threshold. Clustering analysis was performed in MATLAB 2021b (The Mathworks Inc., USA).

### Cluster characterization with clinical assessments

We investigated the assessed clinical, physical and gait temporospatial characteristics in each identified cluster. These include muscle strengths of each cluster globally (overall MMT score) and locally (individual joint MMT score), the distribution of WL and SL, distribution of injury levels, physical descriptives (sex, types of walking aids, cause of injury, presence of spasticity, and WISCI II score), walking speed, step length, and step width in each cluster.

### Cluster characterization using random forest and TreeSHAP

To better understand the gait characteristics of each identified cluster, we used random forest classifiers paired with TreeSHAP. In brief, for each identified cluster, we created a dataset by aggregating gait features of its gait patterns with those of the control group. For each gait pattern, we extracted 27 clinically meaningful gait features across the 9 dimensions (Table A1 in supplementary materials). We labeled the features belonging to the iSCI cluster as 1 while the features belonging to the control group were labeled as 0. We trained a random forest classifier to solve this binary classification problem. We then analyzed SHAP values explained by TreeSHAP from the trained model to determine the predominant characteristics that distinguish the gait patterns within the iSCI cluster from those in the control group.

In all training cases, our data set was likely to be highly skewed, with an imbalanced distribution between classes, i.e., dozens of gait patterns for one cluster versus hundreds of gait patterns for the controls. This imbalance could bias the random forest classifier toward the majority class (the controls), increasing the risk of false negatives when predicting a minority class (a cluster of interest). To address this issue, we used a data augmentation technique called Synthetic Minority Over-sampling Technique (SMOTE) [48] to rebalance the classes rather than simply duplicating samples from the minority class. We then performed a grid search for hyperparameter tuning of each model when training on our rebalanced dataset. Specifically, we performed 10-fold stratified cross-validation, searching over key hyperparameters: number of trees (200, 500, 1000), maximum features considered when splitting nodes (“*sqrt*”, “*log*2”), maximum tree depth (10, 15, 20), and measures for quality of splits (“*gini*”, “*entropy*”, “*logloss*”). Among this parameter space, we identified the optimal configuration that achieved maximal cross-validation accuracy. We performed the training process with Python packages [49].

These “best” models were selected and later explained using TreeSHAP. SHAP analysis summarized the most influential gait features impacting the model’s prediction by ranking their mean absolute SHAP values in descending order.

### Statistical tests

After performing clustering, the assumption of normal distribution was violated in some clusters, especially those with outliers, thus we used nonparametric tests. For continuous and ordinal parameters, we used a Kruskal-Wallis test to determine differences among groups, followed by a post-hoc pairwise comparison with Bonferroni correction for p-values to evaluate the difference between two groups. For categorical parameters, we used a Chi-squared test to determine the difference among groups. We used Spearmans’s rank-order correlation coefficient to determine any possible relationships between two variables.

To compare the difference between the iSCI and control group, we used two-sided independent *t*-tests on continuous parameters and Fisher’s exact test on categorical parameters.

To satisfy the independence assumption for statistical testing, we analyzed gait patterns for each side rather than for each individual. Specifically, we chose a median value to represent each side then conducted statistical tests using these median values as independent representations of each side.

We conducted all statistical tests with commercially-available software (IBM SPSS v. 28.0.0.0). For statistical tests, difference was determined at the $\alpha$ = 0.05 level. In Bonferroni corrections of p values, uncorrected p values were multiplied by the number of comparisons.

## Results

### Participant description

**Table 1:** Demographics and clinical descriptives of the participants

	Group		
	iSCI	Control	p
Number	28	21	
Age	57.8 ± 12.6 yrs	50.4 ± 12.3 yrs	0.053^1^
Sex	18 M/ 10 F	15 M/ 6 F	0.760^2^
BMI	26.5 ± 4.6	24.9 ± 2.8	0.170^1^
Walking aids	Barefoot: 23	Barefoot: 21	
	Shoes: 1		
	Crutches^3^: 1		
	Crutches+^4^: 1		
	Walker+^5^: 2		
Diagnosis	Traumatic: 4		
	Nontraumatic: 24		
Time since injury	7 mths to 32 yrs		
	Median: 3 yrs		
AIS	D: 28		
MLI	Cervical: 0		
	Thoracic: 15		
	Lumbar: 8		
	Sacral: 1		
	Intact: 4		
SLI	Cervical: 2		
	Thoracic: 22		
	Lumbar: 4		
	Sacral: 0		
Sum MMT (/90)	75 ± 10		
WISCI II (/20)	20: 21		
	19: 3		
	16: 1		
	13: 1		
	12: 1		
	9: 1		

BMI is Body Mass Index; AIS is ASIA Impairment Scale (A, B, C, D, E); MLI is Motor Level of Injury; SLI is Sensory Level of Injury; Sum MMT is a summed score of Manual Muscle Test on 9 key muscle groups in both legs (0–90); WISCI II is Walking Index for Spinal Cord Injury (0–20);. Age, BMI, and sum MMT are presented as mean ± standard deviation^1^Two-sided independent t-test; ^2^Fisher’s exact test; ^3^Forearm crutches; ^4^Forearm crutches, shoes and ankle-foot orthosis; ^5^A wheeled walker and shoes

Age, BMI, and sex distribution did not differ between the iSCI and control groups (p > 0.05, Table 1). All participants with iSCI were graded as AIS D. The overall level of walking independence in the iSCI group was relately high; 21 out of 28 participants (75%) reached the highest WISCI II score and 23 out of 28 participants (82%) managed to walk barefoot during the gait analysis session. Most of the participants had nontraumatic SCI (86$\%$). Four participants (S4, S17, S28, S33) had intact motor functions but presented with sensory impairments and/or spasticity (Table A2 in supplementary materials).

### Cluster identification with dynamic time warping and hierarchical agglomerative clustering

**Fig. 3:**
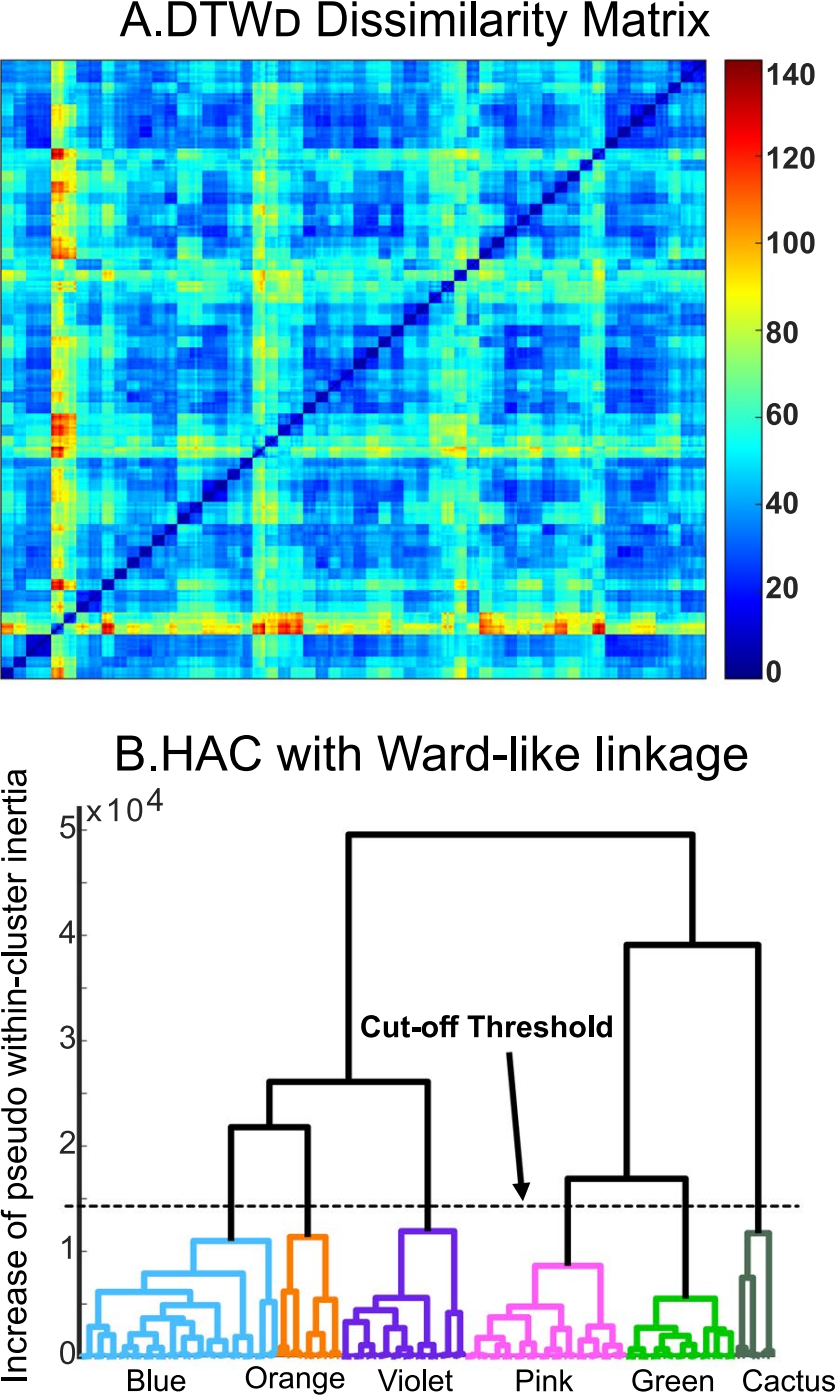
A heatmap representing a dissimilarity matrix based on pairwise computation of $\mathrm {DTW_D}$ distances between any two gait patterns among the 280 patterns. A dendrogram built from HAC with Ward-like linkage on the $\mathrm {DTW_D}$-based dissimilarity matrix, with the cut-off threshold for distinguishing subgroups evident. The vertical axis represents the increase of pseudo within-cluster inertia when merging two partitions

We extracted a total of 280 gait patterns from 28 participants with iSCI and computed all pairwise $\mathrm {DTW_D}$ distances to generate a 280x280 distance matrix. We identified six clusters (Figure 3), and named them non-ordinally as colors: Blue (29$\%$, 80/280 patterns, 16/56 sides), Pink (23$\%$, 65/280 patterns, 13/56 sides), Violet (18$\%$, 50/280 patterns, 10/56 sides), Green (16$\%$, 45/280 patterns, 9/56 sides), Orange (9$\%$, 25/280 patterns, 5/56 sides), and Cactus (5$\%$, 15/280 patterns, 3/56 sides).

**Fig. 4:**
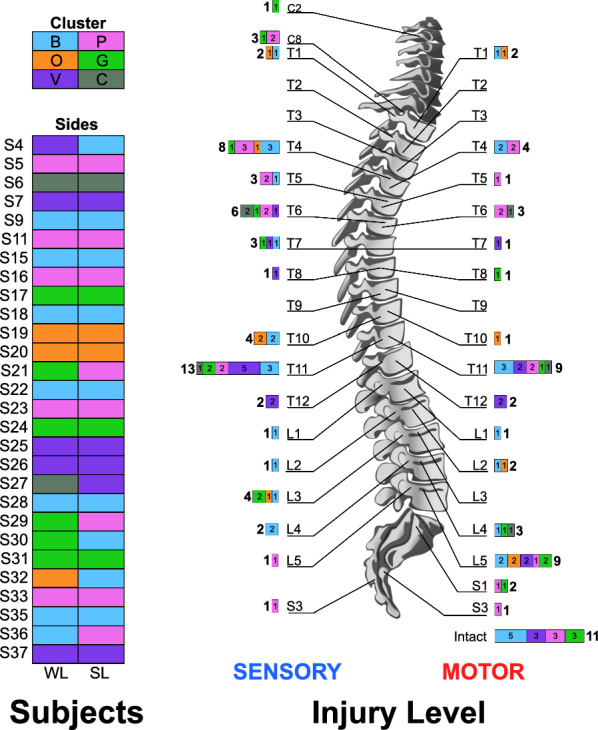
Cluster distribution among the participants with iSCI (left) and across sensory and motor injury levels (right)

**Fig. 5:**
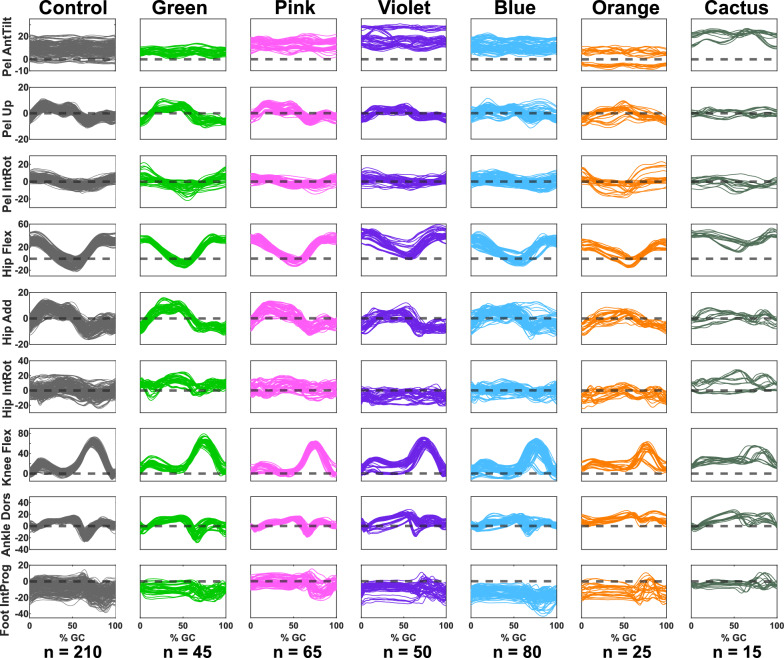
Kinematics plots in the 9 DoFs of the control group and the 6 identified iSCI clusters. The y axis represents the joint angle in degrees, where positive values are indicated in the name of each DoF (Pel AntTilt: Pelvis Anterior Tilt, Pel Up: Pelvis Upward Obliquity, Pel IntRot: Pelvis Internal Rotation, Hip Flex: Hip Flexion, Hip Add: Hip Adduction, Hip IntRot: Hip Internal Rotation, Knee Flex: Knee Flexion, Ankle Dors: Ankle Dorsiflexion, Foot IntProg: Foot Internal Progression)

The distribution of sides, sensory injury level, motor injury level, and gait cluster is shown in Figure 4. Seven iSCI participants exhibited asymmetric gait patterns, with sides belonging to different clusters; in subject S27, the Violet cluster was stronger than the Cactus cluster, in subjects S4, S30, and S32, the Blue cluster was stronger than the Violet, Green, and Orange clusters, respectively, in subject S36, the Pink cluster was stronger than the Blue cluster, and in subjects S21 and S29, the Pink cluster was stronger than the Green cluster. Only participants with sacral motor injury levels were classified in the Pink and Green clusters. Four out of six clusters (Green, Pink, Blue, and Violet) contained sides with intact motor function, which accounted for around 20$\%$ of all sides analyzed.

Figure 5 shows the gait patterns of the derived clusters and of the control group. The gait patterns of the Green and Pink clusters were overall closest to the control group, and the Cactus cluster, furthest.

### Cluster characterization with clinical assessments

**Fig. 6:**
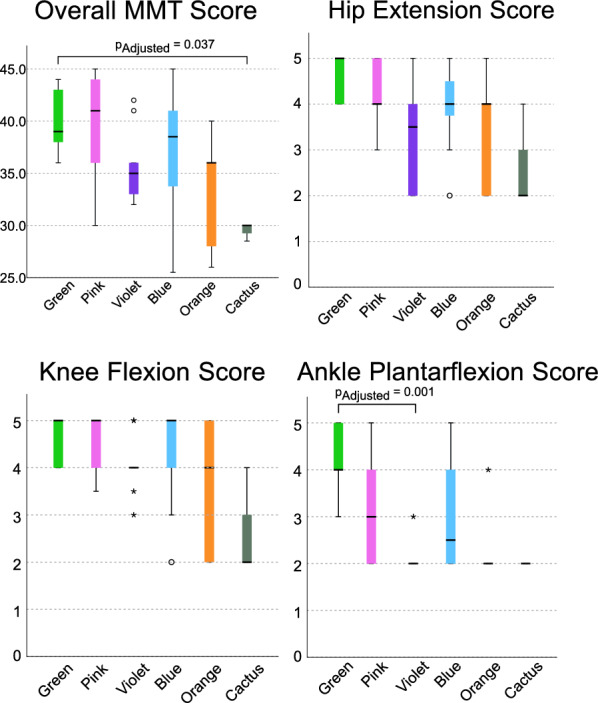
Overall MMT sum score (max 45), hip extension score (max 5), knee flexion score (max 5), and ankle plantarflexion score (max 5) of sides in the six clusters. In the plots, the middle line indicates the median, the box indicates the interquartile range, whiskers indicate the minimum and maximum values that are not outliers, circles (o) indicate outliers, and asterisks (*) indicate extreme outliers

Regarding muscle strength assessment, significant differences among clusters were found in overall MMT sum score ($\mathrm {p = 0.007}$), hip extension score ($\mathrm {p = 0.022}$), knee flexion score ($\mathrm {p = 0.025}$), and ankle plantarflexion score ($\mathrm {p = 0.002}$) (Figure 6). There were no significant pairwise differences between any two clusters in knee flexion and hip extension score (all $\mathrm {p_{Adjusted}} > 0.05$). In overall MMT sum score, there was a significant difference between Green and Cactus clusters ($\mathrm {p_{Adjusted}} = 0.037$). For ankle plantarflexion score, there was a significant difference between Green and Violet clusters ($\mathrm {p_{Adjusted}} = 0.001$).

**Fig. 7:**
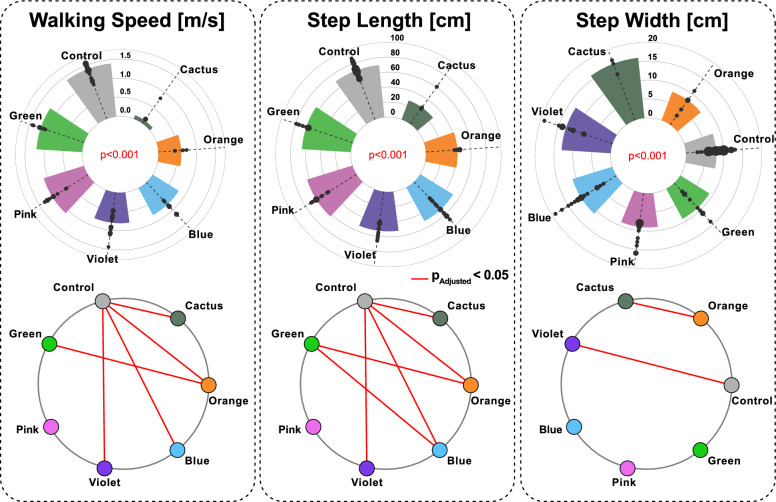
Gait temporospatial parameters in the iSCI clusters and the control group. The group median values were ranked and ordered counter-clockwise. Each black dot represents the value for one limb, with increasing diameter for higher number of limbs with same value. Significant pairwise differences between groups, determined through post-hoc comparisons with Bonferroni correction where the adjusted p value is less than 0.05, are highlighted using red lines connecting the corresponding groups

Significant differences were found among clusters for all gait temporospatial parameters (Figure 7, and Table A3 in supplementary materials). After the control group, walking speed and step length magnitudes in descending order were: Green, Pink, Violet, Blue, Orange, Cactus clusters. Post-hoc comparisons indicated no significant differences in walking speed or step length between controls and the Pink cluster, or between controls and the Green cluster. Magnitude of step width were in descending order: Cactus, Violet, Blue, Pink, Green, Control, and Orange clusters, wherein significant differences were observed between Cactus and Orange clusters, and between controls and the Violet cluster.

**Table 2:** Clinical descriptives in the six identified clusters

	Cluster					
	Green	Pink	Violet	Blue	Orange	Cactus
	n = 45	n = 65	n = 50	n = 80	n = 25	n = 15
Sex	Female	Female	Female	Female	Female	Female
p = 0.009^1^	22$\%$	77$\%$	10$\%$	38$\%$	20$\%$	0$\%$
	Male	Male	Male	Male	Male	Male
	78$\%$	23$\%$	90$\%$	62$\%$	80$\%$	100$\%$
Walking aids	Barefoot	Barefoot	Barefoot	Barefoot	Barefoot	Barefoot
p < 0.001^1^	100$\%$	100$\%$	80$\%$	81$\%$	40$\%$	33$\%$
			Shoes	Crutches+	Walker+	Crutches
			20$\%$	13$\%$	60$\%$	67$\%$
				Walker+		
				6$\%$		
Presence of spasticity	22$\%$	23$\%$	20$\%$	38$\%$	0$\%$	100$\%$
p = 0.054^1^						
WISCI II	20	20	20	20	20	20
p=0.008^2^	100$\%$	100$\%$	60$\%$	69$\%$	40$\%$	33$\%$
			19	19	19	19
			40$\%$	12$\%$	0$\%$	0$\%$
			<19	<19	<19	<19
			0$\%$	19$\%$	60$\%$	67$\%$

^1^Chi-squared test; ^2^Kruskal-Wallis test

Clinical descriptives differed significantly among clusters regarding sex and walking aids, but not for presence of spasticity (Table 2). Regarding sex, five out of six clusters were predominantly male (above 50%), except for the Pink cluster (77% female). Regarding walking aids, Pink and Green clusters were predominantly barefoot. In all clusters, most injuries were nontraumatic. Although no significant difference among clusters was found for the presence of spasticity, it is worth noting that all in the Cactus cluster presented with spasticity, whereas none in the Orange cluster did. WISCI II scores differed among clusters, wherein Pink and Green clusters had maximum WISCI II scores (20/20), reflecting high levels of walking independence.

### Cluster characterization using random forest and TreeSHAP

Balancing the dataset with SMOTE technique improved the performance of the random forest classifiers. With optimal hyperparameters, five out of six models achieved a mean accuracy of 1 in distinguishing each cluster from controls (Table A4 in supplementary materials).

**Fig. 8:**
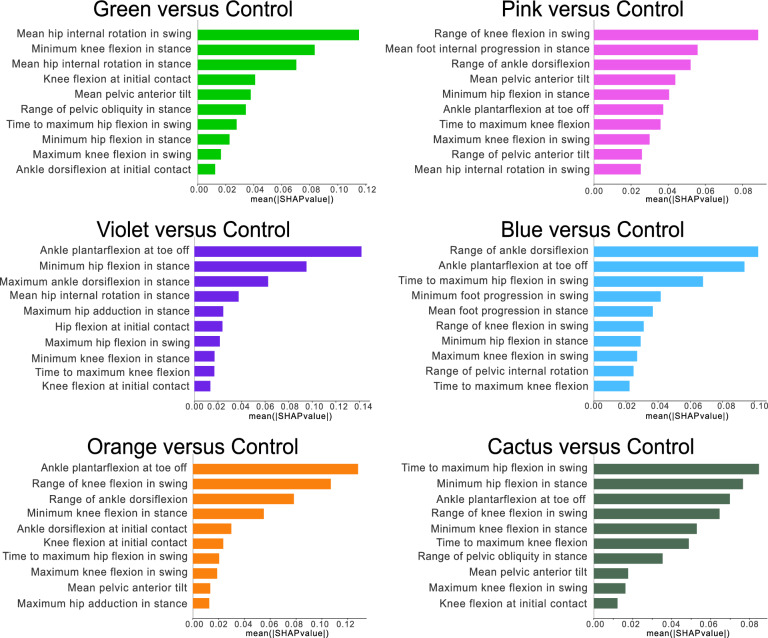
Top 10 gait features in the clusters that most distinguish them from controls in order of descending mean absolute SHAP values. Explanations from TreeSHAP based on random forest classifiers trained on enhanced datasets of each cluster and the controls

- *Green*: The most pronounced gait features that distinguish Green from Control were higher mean hip internal rotation angle during swing, higher minimum knee flexion angle in stance, and higher mean hip internal rotation angle during stance (Figure 8). The mean hip internal rotation angles during swing and stance were higher in Green (median 7$^{\circ }$ internal rotation and 11$^{\circ }$ internal rotation respectively) than in Control (median 4$^{\circ }$ external rotation and 2$^{\circ }$ internal rotation respectively). Minimum knee flexion angle during stance was higher in Green (median 4$^{\circ }$ flexion) than in Control (median 2$^{\circ }$ extension).
- *Pink*: The most pronounced gait features that distinguish Pink from Control were lower range of knee flexion in swing, higher mean foot internal progression in stance, and lower range of ankle dorsiflexion. The ranges of ankle dorsiflexion and knee flexion in swing were lower in Pink (median 24$^{\circ }$ and 61$^{\circ }$ respectively) than in Control (median 28$^{\circ }$ and 67$^{\circ }$ respectively). Mean foot internal progression in stance was higher in Pink (median 3$^{\circ }$ external foot progression) than in Control (median 6$^{\circ }$ external foot progression).
- *Violet*: The most pronounced gait features that distinguish Violet from Control were lower ankle plantarflexion angle at toe-off, higher minimum hip flexion angle in stance, and and higher maximum ankle dorsiflexion angle in stance. Ankle plantarflexion angle at toe-off was lower in Violet (median 3$^{\circ }$ dorsiflexion) than in Control (median 11$^{\circ }$ plantarflexion). Minimum hip flexion angle in stance was higher in Violet (median 5$^{\circ }$ flexion) than in Control (median 10$^{\circ }$ extension). Maximum ankle dorsiflexion angle in stance was higher in Violet (median 22$^{\circ }$ dorsiflexion) compared to Control (median 13$^{\circ }$ dorsiflexion).
- *Blue*: The most pronounced gait features that distinguish Blue from Control were lower range of ankle dorsiflexion, lower ankle plantarflexion angle at toe-off, and later timing of maximum hip flexion in swing. The range of ankle dorsiflexion was lower in Blue (median 21$^{\circ }$) than in Control (median 28$^{\circ }$). Ankle plantarflexion angle at toe-off was lower in Blue (median 1$^{\circ }$ plantarflexion) than in Control (median 11$^{\circ }$ plantarflexion). The timing of maximum hip flexion in swing was later in Blue (median 1.2s) than Control (median 0.9s).
- *Orange*: The most pronounced gait features that distinguish Orange from Control were lower ankle plantarflexion angle at toe-off, lower range of knee flexion in swing, and lower range of ankle dorsiflexion. Ankle plantarflexion angle at toe-off was lower in Orange (median 13$^{\circ }$ dorsiflexion) than in Control (median 11$^{\circ }$ plantarflexion). The ranges of ankle dorsiflexion and knee flexion during swing were lower in Orange (median 15$^{\circ }$ and 39$^{\circ }$ respectively) than in Control (median 28$^{\circ }$ and 67$^{\circ }$ respectively).
- *Cactus*: The most pronounced gait features that distinguish Cactus from Control were later timing of maximum hip flexion in swing, higher minimum hip flexion angle during stance, and lower ankle plantarflexion angle at toe-off. The timing of maximum hip flexion in swing was later in the Cactus (median 3.5s) than Control (median 0.9s) for the Control. Maximum hip extension angle in stance was lower in Cactus (median 25$^{\circ }$ flexion) compared to Control (median 10$^{\circ }$ extension). Maximum ankle plantarflexion angle at toe-off was lower in Cactus (median 5$^{\circ }$ dorsiflexion) than in Control (median 11$^{\circ }$ plantarflexion).

### Correlation analysis

**Fig. 9:**
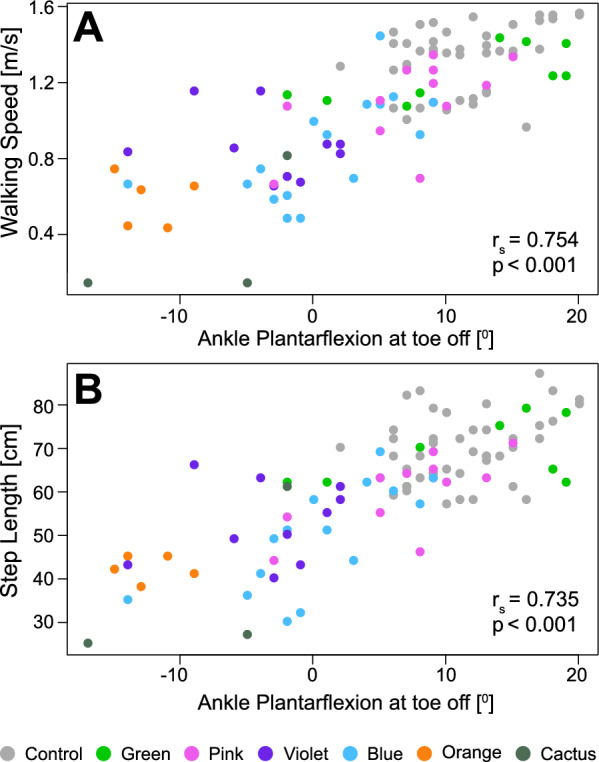
Scatterplots of correlation between ankle plantarflexion angle at toe-off and walking speed (**A**), between ankle plantarflexion angle at toe-off and step length (**B**). Each dot represents one median value of the side belonging to the group of interest

Less ankle plantarflexion at toe-off than Control was identified as the most frequent feature among clusters. SHAP analysis revealed that this gait feature ranked first in Orange and Violet, second in Blue, and third in Cactus. We investigated whether this gait feature correlates to slower walking speed and shorter step length, and found strong, positive correlations between ankle plantarflexion angle at toe-off and walking speed ($r_s$(98) = 0.754, p < 0.001, Figure 9), and between ankle plantarflexion angle at toe-off and step length ($r_s$(98) = 0.735, p < 0.001).

## Discussion

The main finding of this study was the identification of 6 distinct clusters that categorize gait in a heterogeneous iSCI cohort according to their gait patterns. Four clusters (Cactus, Orange, Blue, and Violet) exhibited significantly slower walking speed and shorter step length than controls. This aligns with previous findings [50]. However, the Pink and Green clusters managed to walk barefoot, and exhibited comparable walking speeds and step lengths to controls.

The gait features in the Green and Pink clusters deviated slightly from controls. The hips in the Green cluster tended to rotate internally, and the knees were slightly flexed during midstance. In the Pink cluster, the ranges of ankle dorsiflexion and knee flexion during swing were slightly lower than those in the Control.

The Cactus cluster exhibited persistent knee flexion and lower range of motion at the hip and knee throughout the gait cycle. Spasticity might be a contributing factor to these gait deviations as it was present on all sides in the Cactus cluster. The Orange cluster, on the other hand, had no spasticity at all. The severely restricted knee range of motion in the Orange cluster likely contributed to shorter step lengths. Moreover, the difference in ankle plantarflexion angle at toe-off between the Orange cluster and the Control was large, where 5$^{\circ }$ difference is already considered as clinically significant [51]. The severe gait deficits at the ankle is likely related to plantarflexor weakness; plantarflexor strength on most sides in the Orange cluster and all sides in the Cactus cluster was scored with a 2 (Poor) in MMT, indicating ability to perform plantarflexion without resistance but inability to perform a single leg heel raise. This weakness likely led to a compromised solution, which involved walking more slowly.

Both the Violet and Blue clusters had the most pronounced gait deviations at the hip and the ankle. The hips in the Violet cluster were not fully extended during gait, related to the increased anterior pelvic tilt. In the Blue cluster, the timing to maximum hip flexion in swing was later than the Control, although not as much as in the Cactus cluster. In the Violet cluster, the excessive dorsiflexion during stance is related to the persistent knees flexion. The Blue cluster exhibited low plantarflexion angle in pre-swing, as did the Violet cluster. The strength of the plantarflexors varied considerably in the Blue cluster, from MMT scores of 2 (Poor) to 5 (Normal), median 2–3, and were slightly stronger than the Violet cluster, yet still insufficient for full functional capability. Similar to the Orange and Cactus clusters, inadequate plantarflexion strength in the Blue and Violet clusters likely contributed to slower walking speed and shorter step length.

Out of a total of 27 investigated features, ten appeared in the three most influential features that distinguished the clusters from Control (Table A5 in supplementary materials). Among these, hip features appeared 6 times, knee features appeared 3 times, and ankle and foot features appeared 9 times. Notably, no pelvis features were identified as important by the SHAP analysis. This finding suggests that hip and ankle joint kinematics may contain the most valuable information for distinguishing the gait patterns of the iSCI clusters from normal gait and/or that pelvis deviation occurs in combination with other joint deviations. Furthermore, ankle plantarflexion at toe-off was consistently important across the Pink, Violet, Blue and Cactus clusters, signifying that lower plantarflexion at toe-off is as a shared gait deficit in these clusters, and suggesting its influence in the lower walking speed and shorter step length, as supported by the strong, positive correlations between both walking speed and step length and ankle plantarflexion angle at toe-off.

This shared compensatory strategy of slower walking speed observed across most of our iSCI clusters aligns with reports by Pepin et al. [14]. However, unlike their study, we proved that not all clusters exhibited slower walking speeds as those in high-functioning clusters (Green and Pink) demonstrated walking speeds comparable to control participants. Previous work by Krawetz and Nance clustered iSCI participants based on injury level (cervical, thoracic, and lumbar) [13]. However, our analysis revealed that the identified clusters were distributed across all investigated injury levels (Figure 4), indicating that injury level alone does not determine gait pattern differences. Participants with higher-level injuries did not necessarily exhibit more altered gait patterns than those with lower-level injuries.

The selection of five gait cycles per limb balanced capturing natural gait variability with feasible data collection for individuals with iSCI, who may experience fatigue during prolonged walking. We aimed to extract five cycles per leg from five different trials, which was possible in 12/28 participants (S17, S20, S22, S23, S24, S27, S28, S29, S30, S31, S36, and S37). Due to data availability, we extracted the five trials per side from 4 different trials in 7/28 participants (S05, S11, S21, S25, S32, S33, and S35), from 3 different trials in 2/28 participants (S09, S19), and from 2 different trials in 1/28 participants (S06). Although some cycles were collected within the same trial, the consistent clustering of all five cycles from each participant’s limb within the same cluster demonstrates that our findings reflect stable, individual-specific gait characteristics rather than trial-specific variations. This robust clustering supports the validity of our cluster identification and suggests that step-to-step dependencies did not substantially impact the identification of distinct gait phenotypes in the iSCI clusters.

**Fig. 10:**
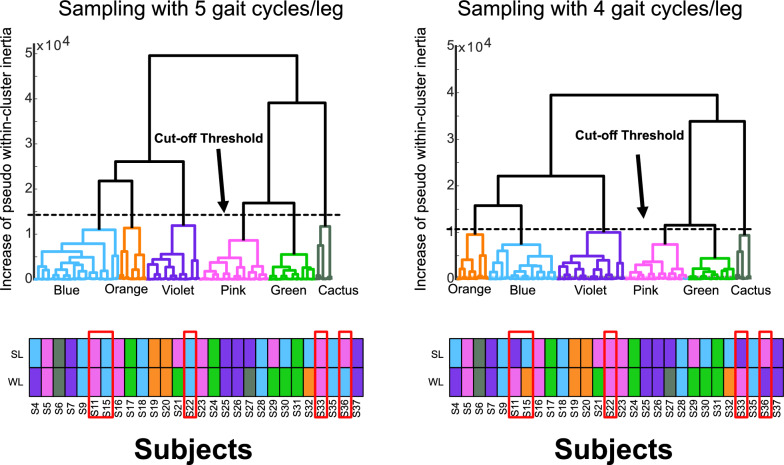
Dendrogram and cluster distribution comparing 5 gait cycles per leg (left) and 4 gait cycles per leg (right). Red rectangles highlight participants with different cluster assignments between the two sampling conditions

To assess the robustness of our clustering approach to sampling variability, we conducted an ablation study by removing one gait cycle per leg and repeating the clustering analysis using HAC with DTW. The analysis with 4 gait cycles per leg identified 6 clusters, with 50 out of 56 limbs (89%) maintaining consistent cluster distribution (changes observed in 6 limbs: S11 SL, S15 WL, S22 SL, S22 WL, S33 SL, and S36 WL; Figure 10). Notably, the Green and Cactus clusters remained stable when reducing the number of gait cycles, demonstrating robustness against sampling variability. Among the affected limbs, transitions occurred primarily from higher-functioning clusters (Pink and Blue) to lower-functioning ones (Orange or Violet), suggesting that sampling variability may influence the detection of subtle functional differences between clusters. This analysis demonstrates that while our clustering approach shows promise with strong overall stability, continued refinement of sampling protocols will enhance the reliability of gait phenotype identification.

The results in post-hoc comparisons revealed no significant differences between clusters in hip extension and knee flexion strength subscores. A possible explanation for this might be that the investigated iSCI group was in general highly functioning. There were no sides having scores of 0 or 1. We speculate that with a larger study population with a wider range of walking function, we might see more differences in specific muscle group strengths between clusters.

There are several methods to speed up the computation of DTW for big datasets such as lower bounding nearest search [52, 53] or early abandoning techniques [25]. However, since the data set was small in this study, there was no need for those methods as the computing time was acceptable (approximately 24 min). The matrix is symmetric, so one can compute half of the $\textrm{DTW}$ distances and mirror them across the diagonal to reduce computation time. For data preparation, performing Z-normalization locally would also remove mean values in each gait pattern which were also important in determining dissimilarity among investigated patterns. Therefore, we computed Z-normalization on all derived kinematics data.

The choice of linkage method is the most decisive factor in HAC. An inconsistent linkage can lead to distorted dendrograms that undermine interpretation and discovery of natural hierarchical relationships. Ward linkage is commonly favored for HAC by minimizing within-cluster variance at each iteration, yielding compact, evenly sized clusters with high internal consistency. Since Ward linkage relies on Euclidean distances in determining merging costs in each iteration, it needs some modifications for compatibility with our DTW-based dissimilarity matrix. Therefore, we implemented the Ward-like HAC method to enable the use of a matrix built from non-Euclidean distances.

HAC inherently requires balancing the number of clusters against within cluster inconsistency. There are no definitive thresholds. As cluster analysis is essentially an exploratory approach, the interpretation of the resulting hierarchical structure is context-dependent. Too high a threshold will fail to identify clusters, and too low a threshold will identify an excessive number of clusters. For instance, in the current study, a higher threshold (30,000) would have resulted in 3 unequally distributed clusters with poor internal cohesion. A moderately higher threshold (20,000) would have resulted in a merged Pink-Green cluster with notably higher inconsistency than other clusters. We selected the threshold (just below 15,000) since it resulted in distinct clusters of similar size with strong internal consistency.

In this study, random forests were chosen, in part as it is a frequently used machine learning model across various topics such as finance [54], environment [39], and healthcare [55], and in part due to its compatibility with TreeSHAP. Moreover, random forests are also known to achieve high accuracy compared to other machine learning algorithms and to handle complex and high-dimensional data sets efficiently, particularly tabular data [56].

While we exhaustively invited potential patients with iSCI from the neurorehabilitation clinic over a period of nearly 2 years, and had few dropouts and refusals to participate, our sample size can still be considered as relatively small, consisting of 28 individuals with iSCI. The sample size in the current study indeed limits its generalizability across the broader iSCI population. Previous gait studies on persons with iSCI included larger cohorts, such as Werner et al. [21] who studied 66 persons and extracted gait parameters from inertial measurement units, and Basiratzadeh et al. [20] who conducted a retrospective study of 334 persons. Neither study performed comprehensive 3D gait analysis.

The iSCI cohort in this study was highly functional, with 21 out of 28 participants achieving the maximum score on the WISCI II score. The total sides of Cactus and Orange clusters represented only approximately 14% of the total sides investigated. As a result, the number and characteristics of the clusters may differ if the analysis incorporates more data from subjects with relatively low mobility.

We treated the left and right sides independently. An argument for treating both sides together can be made; the two sides are not entirely independent observations. Likewise, an argument for treating them independently can also be made; due to the highly asymmetric strength distribution in the population, an average of sides represents neither the stronger nor the weaker side and instead attenuates distinguishing characteristics of each. If sides are treated together, the dimensional representation is higher, and requires sufficient data to support the increased complexity. It is unclear how DTW would perform on such high dimensional data. We fixed the weights and window sizes in computing DTW distances for simplicity. Tuning these may have enabled better gait pattern classification, but this was outside the scope of this study.

Future work could aim to recruit a more diverse sample of individuals with low ambulation ability due to iSCI. Moreover, future work should explore faster and more efficient methods for DTW distance calculation. This could help address potential limitations in computing the full dissimilarity matrix as the number of gait comparisons grows with additional subjects. Future work should incorporate systematic validation of cluster stability across different cycle selection strategies and sample sizes to ensure robust and reproducible gait phenotype identification. Further analyses with joint kinetics and ground reaction forces are also warranted.

The methods we describe here are intended to complement traditional clinical assessments and gait analysis, and can enable entirely new and more nuanced perspectives that clinians can incorporate into individualized treatment planning. As clusters display distinguishing gait features and commonalities in clinical descriptives and assessments, they can be a context to tailor therapy or interventions that address the distinguishing gait impairments of each cluster. Physiotherapists might recommend targeted strength training and stretching exercises for individuals in the Orange cluster, focusing on strengthening the plantarflexors when possible. Persons in the Green cluster might also benefit from exercises aimed at strengthening the gluteal muscles, when possible. In cases where targeted exercise is not possible after nerve damage, external supports such as orthoses or exoskeletons could be used to compensate for underlying muscle weakness. Follow-up studies are warranted to investigate the potential of cluster-dependent intervention plans for improved outcome.

## Conclusion

Findings from this study demonstrate the ability to characterize the overall gait heterogeneity in a population of persons with iSCI into distinct clusters using data-driven approaches. The synergistic integration of clinical experience and data-driven insight can enable discoveries that may be missed using either approach alone.

## Additional file

Supplementary file 1.

Supplementary file 2.

## Data Availability

Due to Swedish and EU personal data legislation, the raw data set is not publicly available but is available from the corresponding author upon appropriate request. Any sharing of data will be regulated via a data transfer and user agreement with the recipient. The post-processed data set and code are available at: https://github.com/Minh309/iSCI-gait-stratification

## Abbreviations

6MWT: Six-Minute Walk Test
10mWT: Ten-meter Walk Test
iSCI: Incomplete Spinal Cord Injury
ASIA: American Spinal Injury Association
AIS: ASIA Impairment Scale
BMI: Body Mass Index
DoF: Degrees of Freedom
DTW: Dynamic Time Warping
$\mathrm {DTW_D}$: Dependent Dynamic Time Warping
$\mathrm {DTW_I}$: Independent Dynamic Time Warping
HAC: Hierarchical Agglomerative Clustering
MLI: Motor Level of Injury
MMT: Manual Muscle Test
SCI: Spinal Cord Injury
SL: Stronger Leg
SLI: Sensory Level of Injury
SHAP: SHapley Addictive exPlanations
SMOTE: Synthetic Minority Oversampling Technique
WISCI II: Walking Index for Spinal Cord Injury II
WL: Weaker Leg
