# Supplementary material for: Gait pattern stratification in persons with incomplete spinal cord injury: a data-driven approach
Source: J Neuroeng Rehabil. 2026 Feb 26;23:108. doi: 10.1186/s12984-026-01896-w (PMC13041457; doi:10.1186/s12984-026-01896-w)
Supplement: Supplementary file 1 [file 12984_2026_1896_MOESM1_ESM.pdf]

Supplementary materials

Table A1 Descriptions of kinematics features extracted from each gait pattern

Index	Description
1	Mean pelvic anterior tilt [°]
2	Range of pelvic anterior tilt [°]
3	Mean pelvic internal rotation [°]
4	Range of pelvic internal rotation [°]
5	Maximum pelvic obliquity in stance [°]
6	Range of pelvic obliquity in stance [°]
7	Hip flexion at initial contact [°]
8	Minimum hip flexion in stance [°]
9	Maximum hip flexion in swing [°]
10	Time to maximum hip flexion in swing [s]
11	Maximum hip adduction in stance [°]
12	Maximum hip abduction in swing [°]
13	Range of hip abduction in stance [°]
14	Mean hip rotation in stance [°]
15	Mean hip rotation in swing [°]
16	Knee flexion at initial contact [°]
17	Minimum knee flexion in stance [°]
18	Maximum knee flexion in loading response [°]
19	Maximum knee flexion in swing[°]
20	Time to peak knee flexion [s]
21	Range of knee flexion in swing [°]
22	Ankle dorsiflexion at initial contact [°]
23	Maximum ankle dorsiflexion in stance[°]
24	Range of ankle dorsiflexion[°]
25	Ankle plantarflexion at toe-off [°]
26	Mean foot internal progression in stance [°]
27	Minimum foot internal rotation in swing [°]

Table A2 Characteristics of the participants in iSCI group

ID	Age [years]	Sex [M/F]	Height [m]	Weight [kg]	TSI [months]	MLI	SLI	Spasticity [/60]	Aids	WISCI II
S04	58	F	1.57	86.9	7	Intact	T5	0	Barefoot	20
S05	53	F	1.76	111.8	7	T5	T5	0	Barefoot	20
S06	61	M	1.75	73.7	17	T6	T6	15	Crutches	16
S07	65	M	1.79	114.5	24	T11	T11	9	Barefoot	20
S09	71	F	1.54	72.0	36	T11	T11	0	Barefoot	20
S11	53	M	1.85	103.1	168	T4	T4	2	Barefoot	20
S15	73	M	1.79	95.3	18	L4	T10	0	Barefoot	20
S16	51	F	1.60	64.0	42	T6	T6	0	Barefoot	20
S17	32	M	1.87	83.6	24	Intact	T6	0	Barefoot	20
S18	57	M	1.69	69.8	384	T4	T4	1	Barefoot	19
S19	69	M	1.84	95.0	120	T10	T10	0	Walker+	9
S20	74	M	1.65	95.0	24	L5	T4	0	Barefoot	20
S21	69	F	1.71	58.7	12	L5	T4	9	Barefoot	20
S22	61	F	1.59	52.9	180	L1	L1	0	Crutches+	12
S23	49	F	1.68	62.4	18	S1	L5	0	Barefoot	20
S24	71	M	1.85	91.6	24	L4	L3	0	Barefoot	20
S25	75	M	1.76	67.4	36	T12	T12	0	Barefoot	20
S26	73	M	1.78	92.0	108	L5	T11	0	Shoes	19
S27	53	M	1.83	70.6	30	T11	T11	7	Barefoot	20
S28	30	M	1.89	97.0	36	Intact	T4	5	Barefoot	20
S29	73	F	1.63	51.9	144	T11	T11	1	Barefoot	20
S30	42	M	1.73	74.8	204	L5	T7	0	Barefoot	20
S31	54	M	1.81	80.8	10	T8	C2	0	Barefoot	20
S32	63	F	1.67	68.1	84	T1	T1	0	Walker+	13
S33	56	F	1.63	68.1	16	Intact	C8	0	Barefoot	20
S35	35	M	1.75	80.0	72	L2	L3	4	Barefoot	20
S36	45	M	1.76	90.0	60	T11	T11	15	Barefoot	20
S37	68	M	1.75	95.7	144	T7	T7	0	Barefoot	19

All participants were evaluated as AIS D. TSI - Time Since Injury; MLI - Motor Level of Injury; SLI - Sensory Level of Injury; Spasticity - summed modified Ashworth scores on all joints in both sides; AIS - ASIA Impairment Scale

Table A3 Spatiotemporal performances of the six identified clusters and controls. The values are presented as Median [Q1, Q3]. The values significantly differed from controls from post-hoc comparisons are highlighted.

	Walking Speed (m/s)	Step Length (cm)	Step Width (cm)
Controls	1.36 [1.23, 1.47]	70 [64, 75]	8 [6, 10]
Green	1.23 [1.12, 1.41]	65 [62, 76]	9 [4, 11]
Pink	1.10 [1.00, 1.26]	63 [55, 64]	9 [8, 13]
Violet	**0.84 [0.69, 0.94]**	**52 [43, 61]**	**13 [10, 14]**
Blue	**0.83 [0.61, 1.08]**	**51 [37, 61]**	11 [7, 14]
Orange	**0.63 [0.43, 0.69]**	**42 [39, 45]**	8 [4, 9]
Cactus	**0.14 [0.14, 0.81]**	**27 [25, 61]**	16 [13, 17]

Table A4 Optimal hyperparameters for each random forest classifier after 10-fold stratified cross-validation

Model	Dataset	Optimal Hyperparameters				Accuracy
		Criterion	Max Depth	Max Features	Number of Estimators	
Blue vs Control	Ordinary	entropy	10	log2	200	0.983
	Enhanced	gini	10	sqrt	200	0.998
Orange vs Control	Ordinary	gini	10	log2	500	0.998
	Enhanced	gini	10	sqrt	200	1.000
Violet vs Control	Ordinary	gini	10	sqrt	200	1.000
	Enhanced	gini	10	sqrt	200	1.000
Pink vs Control	Ordinary	entropy	10	sqrt	500	0.982
	Enhanced	gini	15	sqrt	200	1.000
Green vs Control	Ordinary	entropy	10	sqrt	200	0.996
	Enhanced	gini	10	sqrt	200	1.000
Cactus vs Control	Ordinary	gini	10	sqrt	500	1.000
	Enhanced	gini	10	sqrt	200	1.000

Table A5 Gait kinematics features in Controls and six identified clusters. The values are presented as Median [Q1, Q3].

Gait features	Control	Cactus	Orange	Violet	Blue	Green	Pink
Pel_Ant_Tilt_mean[°]	9 [6, 13]	22 [20, 23]	5 [-5, 8]	17 [11, 19]	10 [8, 14]	7 [4, 8]	14 [10, 16]
Pel_Ant_Tilt_range[°]	3 [3, 4]	5 [5, 11]	5 [4, 6]	5 [3, 5]	5 [5, 6]	4 [3, 4]	5 [4, 5]
Pel_Int_Rot_mean[°]	0 [-1, 1]	-2 [-4, 0]	-1 [-3, 2]	0[0, 1]	2 [-3, 3]	0 [-2, 2]	0 [-2, 1]
Pel_Int_Rot_range[°]	10 [8, 12]	9 [9, 11]	12 [6, 24]	9 [6, 12]	10 [5, 13]	11 [8, 13]	10 [7, 11]
Pel_Obl_Stance_max[°]	6 [5, 8]	4 [2, 4]	5 [3, 5]	5 [4, 6]	6 [4, 6]	6 [5, 8]	6 [6, 8]
Pel_Obl_Stance_range[°]	11 [9, 12]	4 [4, 5]	8 [8, 13]	9 [7, 11]	8 [7, 11]	14 [13, 14]	11 [10, 12]
Hip_Flex_IC[°]	33 [28, 35]	39 [39, 43]	24 [18, 29]	42 [37, 44]	31 [29, 34]	32 [30, 33]	35 [32, 37]
Hip_Flex_Stance_min[°]	-10 [-14, -4]	**25 [19, 26]**	-1 [-12, -1]	**5 [3, 10]**	-3 [-6, 1]	-8 [-12, -7]	-4 [-6, -2]
Hip_Flex_Swing_max[°]	33 [29, 37]	40 [40, 45]	27 [18, 28]	42 [37, 44]	33 [31, 36]	34 [31, 37]	36 [31, 37]
Hip_Flex_Swing_max_time[s]	0.9 [0.9, 1.0]	**3.5 [2.5, 3.6]**	1.1 [1.1, 1.9]	1.1 [1.1, 1.1]	**1.2 [1.0, 1.2]**	1.0 [1.0, 1.1]	1.0 [0.9, 1.1]
Hip_Add_Stance_max [°]	9 [7, 10]	4 [2, 6]	6 [3, 6]	6 [2, 7]	6 [6, 9]	9 [8, 11]	8 [6, 11]
Hip_Abd_Swing_max[°]	9 [7, 11]	3 [2, 5]	6 [6, 9]	10 [8, 11]	9 [7, 10]	9 [8, 10]	10 [7, 11]
Hip_Abd_Swing_range[°]	6 [5, 8]	4 [4, 6]	5 [3, 8]	5 [4, 8]	6 [4, 8]	7 [5, 7]	7 [6, 8]
Hip_Int_Rot_Stance_mean[°]	2 [-3, 7]	13 [6, 15]	-2 [-10, 0]	-7 [-8, -5]	2 [-1, 4]	**11 [9, 14]**	6 [-1, 8]
Hip_Int_Rot_Swing_mean[°]	-4 [-7, 0]	7 [5, 12]	-4 [-8, -3]	-7 [-9, -5]	-1 [-4, 1]	**7 [4, 10]**	2 [-4, 5]
Knee_Flex_IC[°]	0 [-2, 1]	15 [13, 17]	10 [8, 12]	10 [2, 13]	2 [-1, 6]	7 [3, 10]	1 [1, 2]
Knee_Flex_Stance_min[°]	-2 [-3, 1]	15 [13, 17]	8 [8, 8]	9 [-1, 12]	0 [-3, 1]	**4 [3, 6]**	-1 [-1, 1]
Knee_Flex_LR_max[°]	17 [15, 20]	29 [25, 31]	23 [18, 26]	26 [17, 28]	19 [8, 22]	23 [18, 24]	18 [13, 20]
Knee_Flex_Swing_max[°]	63 [61, 64]	43 [36, 49]	49 [44, 56]	62 [60, 66]	57 [51, 63]	67 [65, 67]	59 [57, 62]
Knee_Flex_max_time[s]	0.8 [0.7, 0.8]	2.9 [2.0, 3.0]	0.9 [0.9, 1.4]	0.9 [0.8, 1.0]	0.9 [0.8, 1.0]	0.8 [0.8, 0.9]	0.8 [0.8, 0.9]
Knee_Flex_Swing_range[°]	67 [62, 69]	24 [20, 32]	**39 [27, 46]**	57 [50, 67]	57 [47, 65]	63 [49, 65]	**61 [60, 63]**
Ankle_Dors_IC[°]	1 [-1, 3]	0 [-1, 2]	10 [7, 11]	3 [2, 5]	-1 [-2, 4]	0 [-10, 1]	0 [-2, 2]
Ankle_Dors_Stance_max[°]	13 [11, 15]	21 [18, 23]	20 [16, 23]	**22 [16, 23]**	15 [12, 17]	12 [12, 15]	12 [11, 15]
Ankle_Dors_range[°]	28 [26, 32]	26 [23, 28]	**15 [14, 19]**	24 [22, 26]	**21 [20, 22]**	30 [25, 34]	**24 [21, 26]**
Ankle_Plan_TO[°]	11 [8, 15]	**-5 [-11, -4]**	**-13 [-14, -11]**	**-3 [-5, 2]**	**1 [-2, 5]**	14 [7, 18]	9 [5, 9]
Foot_Int_Pro_Stance_mean[°]	-6 [-12, -3]	-3 [-4, -3]	-14 [-16, -8]	-8 [-11, -5]	-14 [-17, -11]	-8 [-13, -8]	**-3 [-5, 3]**
Foot_Int_Pro_Swing_min[°]	-15 [-22, -13]	-10 [-12, -9]	-14 [-23, -13]	-14 [-23, -14]	-23 [-26, -20]	-16 [-19, -14]	-14 [-15, -10]
